# Eugenol Attenuates Transmissible Gastroenteritis Virus-Induced Oxidative Stress and Apoptosis Via ROS-NRF2-ARE Signaling

**DOI:** 10.3390/antiox11091838

**Published:** 2022-09-18

**Authors:** Kang Wang, Yan Tang, Xiu Wu, Hongmin Liang, Daiwen Chen, Bing Yu, Jun He, Xiangbing Mao, Zhiqing Huang, Hui Yan, Aimin Wu, Yuheng Luo, Ping Zheng, Jie Yu, Huifen Wang, Junqiu Luo

**Affiliations:** 1 Institute of Animal Nutrition, Sichuan Agricultural University, Chengdu 611130, China; 2 Key Laboratory of Animal Disease-Resistant Nutrition, Chengdu 611130, China

**Keywords:** transmissible gastroenteritis virus, coronavirus, eugenol, oxidative stress, reactive oxygen species, apoptosis, weaned pigs

## Abstract

Transmissible gastroenteritis virus (TGEV), a coronavirus that causes severe diarrhea due to oxidative stress in the piglet intestine, is a major cause of economic loss in the livestock industry. However, limited interventions have been shown to be effective in the treatment of TGEV. Here, we demonstrate the therapeutic activity of eugenol in TGEV-induced intestinal oxidative stress and apoptosis. Our data show that eugenol supplementation protects intestine and IPEC-J2 cells from TGEV-induced damage. Mechanistically, eugenol reduces TGEV-induced oxidative stress in intestinal epithelial cells by reducing reactive oxygen species levels. Interestingly, eugenol also inhibits TGEV-induced intestinal cell apoptosis in vitro and in vivo. In conclusion, our data suggest that eugenol prevents TGEV-induced intestinal oxidative stress by reducing ROS-mediated damage to antioxidant signaling pathways. Therefore, eugenol may be a promising therapeutic strategy for TGEV infection.

## 1. Introduction

The variability of coronaviruses and their cross-species spreading signature are thorny issues [1]. TGEV, a coronavirus with a length of 28.5 kb, has become a health threat to the global swine industry, and is the main pathogen causing swine gastroenteritis [2]. Vomiting, severe diarrhea and dehydration are the main clinical symptoms of TGEV infection [3]. It is reported that the mortality rate of TGEV-infected piglets at two weeks of age is up to 100% [4]. Even after the widespread use of vaccines, TGEV infection continues to cause substantial economic losses to the global pig industry [5]. Despite the health impact of the virus on pigs, unfortunately, drugs with evidence-based efficacy for TGEV and its associated complications are limited.

In the current literature, the harmful effects of TGEV are mainly due to oxidative stress caused by virus [6,7], but the exact molecular mechanism remains to be further studied. Oxidative stress is an imbalance between the production of oxidative substances and antioxidant defense in the body, and the main culprit of oxidative stress is ROS [8,9,10]. A large amount of evidence supports that one of the main causes of biological damage caused by TGEV is the generation of ROS [11]. In addition, ROS is an important inducer of apoptosis. When ROS accumulates in large quantities, intrinsic apoptosis will be initiated [12,13]. Oxidative stress and consequent apoptosis are the main features of virus-induced intestinal injury [14]. A previous study showed that TGEV infection could induce increased ROS levels in IPEC-J2 cells, leading to the occurrence of intestinal cell apoptosis [15].

Natural products provide effective resources for the discovery of potentially therapeutic drugs [16]. It has been shown that plant extracts from green barley (*Hordeum vulgare*) [17] and creosote bush (*Larrea tridentata*) [18] significantly increase the viability of human cells undergoing aggressive oxidative stress. Many natural polyphenols inhibit ROS production and interfere with the interaction with the Keap1-Nrf2 signaling pathway to maintain the physical balance of the body [19,20]. Eugenol is a natural plant essential oil with strong antioxidant properties [21]. Eugenol, also called clove oil, is the main constituent of extracted oil from clove (*Syzygium aromaticum* (L.)). Previous literature has shown that eugenol can achieve antioxidant effects by inhibiting intracellular levels of ROS, H_2_O_2_ and NO [22]. A similar result has also been shown in the work of Chen et al., indicating that eugenol attenuates colitis by activating the intestinal Keap1-Nrf2 signaling pathway to reduce oxidative stress [23]. The Keap1-Nrf2-ARE signaling pathway is the most important endogenous antioxidant pathway. Nrf2 is activated under oxidative conditions and induces numerous antioxidative genes and proteins to alleviate damage to cells, tissues, and organs [24,25]. Therefore, the Keap1-Nrf2-ARE signaling pathway is expected to significantly impact the prevention and treatment of the virus-induced intestinal oxidative injury. However, little is known about the physiological function of eugenol in viral infection. More importantly, no studies have demonstrated the critical regulatory role of eugenol in regulating oxidative stress during TGEV infection.

Therefore, this study focused on the effects of eugenol on the antioxidant capacity of intestinal epithelial cells of weaned piglets infected with TGEV, and reveals the underlying mechanism. Our results suggested that eugenol protects intestinal epithelial function by alleviating TGEV-induced oxidative stress and apoptosis, which may be related to the inhibition of TGEV-mediated ROS production.

## 2. Materials and Methods

### 2.1. Reagents

We obtained eugenol (≥98%, W246719) from Sigma-Aldrich. N-Acetyl-L-cysteine (NAC, HY-B0215) was purchased from MedChemExpress.

### 2.2. Virus, Cell Culture, and Treatment

TGEV TS strain (GenBank accession number: HQ462571.1) was preserved in our laboratory [26]. IPEC-J2 cell was obtained from ATCC. IPEC-J2 cells were cultured in DMEM/F12 with 10% FBS and 1% penicillin–streptomycin. Cells were placed in the 37 °C incubator with 5% CO_2_. The cells were pretreated with eugenol (200 μM) or NAC (5 mM) for 1 h and then infected with TGEV (MOI = 1).

### 2.3. Experimental Design and Diet

Twenty-one-day-old Duroc×Landrace×Yorkshire weaned piglets were acquired commercially from a pig farm (Mianyang, Sichuan, China). Piglets were divided into four groups: (1) control piglets; (2) eugenol piglets (400 mg/kg eugenol); (3) TGEV-infected piglets (model); (4) eugenol + TGEV-infected piglets (model + 400 mg/kg eugenol). The piglets were fed basal diets as shown in Table 1, and eugenol was additionally added to the diet. On day 15, all piglets were gavaged and the gavage was carried out by specialists. Briefly, 2.8 × 10^9^ PFU TGEV (TCID50 = 10^−6.67^/100 μL) was administered by gavage to piglets in the two infected groups, and the same dose of homologous medium was administered to piglets in the two uninfected groups. In order to avoid the death of piglets, all piglets were executed on day 18 (date of peak piglet diarrhea) to collect samples.

### 2.4. Sample Collection

On the 18th day of the experiment, blood was collected from the anterior vena cava of the experimental pigs. Whole blood was placed in the blood collection tubes and left to rest at room temperature for 30 min. The samples were centrifuged at 3000 r/min for 10 min and placed in 200 μL eppendorf tubes. The isolated serum was then stored at −20 °C. After the blood collection, the experimental pigs were euthanized. The middle section of piglet jejunum was cut, rinsed with normal saline, and dried with filter paper. The mucosal samples were scraped with glass slides and stored in sterile cryovials and stored at −80 °C.

### 2.5. Serum Antioxidant Indexes

Serum total antioxidant capacity (T-AOC) and malondialdehyde (MDA) concentrations were tested using the commercial kits (Nanjing Jiancheng Bioengineering Institute, Nanjing, China) following product instructions.

### 2.6. Western blotting and RT-PCR

After lysis in RIPA buffer at 4 °C, the protein concentration of the intestinal tissues and cells samples was determined using the BCA protein assay kit (Thermo Fisher, MA, USA, 23225). Western blotting was performed using primary antibodies against Keap1 (1:1000, proteintech, 10503-2-AP), Nrf2 (1:1000, proteintech, 16396-1-AP), HO-1 (1:1000, proteintech, 10701-1-AP), NQO1 (1:1000, proteintech, 67240-1-Ig), BAX (1:500, Santa Cruz, CA, USA, sc-7480), and β-actin (1:2000, CST, 3700). Image Lab 5.1 was used to perform the immunoblotting study quantification.

The extraction of total RNA from cells or tissue homogenates using Trizol reagent (Takara Bio, Dalian, China, 9109) was performed according to the manufacturer’s manual. Prime Script TM RT kit (Takara Bio, RR047A) was used to synthesize cDNA. The primer sequences are shown in Table 2. The 2^−ΔΔCT^ method was used to calculate the relative expression of genes.

### 2.7. Intestinal Epithelial Cell Apoptosis and ROS Level Detection

Jejunum mucosal samples of piglets were cut into pieces in PBS at 4 °C with surgical scissors and centrifuged at 300 g for 5 min. The cell pellets were digested with trypsin for 5 min and fully shaken on a shaker. After centrifugation, the cell pellets were re-suspended with PBS to prepare cell suspension. The suspension was filtered by 70 μm cell sieve, and the cell concentration was adjusted to 1 × 10^6^ cells/mL with PBS.

Next, 100 μL cell suspension was mixed with 5 μL Annexin-V and 5 μL PI (BD, 556547), and then stained for 15 min at room temperature, followed by centrifugation at 300 g for 5 min at 4 °C. Cell precipitate was suspended with 500 μL PBS. Assays were performed by flow cytometry (FACSVerse, BD Biosciences, East Rutherfor, NJ, USA) and analyzed by FlowJo 10.0.7 software. Total apoptotic cells were defined as the sum of the early (Q3) and late (Q2) apoptotic subpopulations [27].

DCFH-DA (Solarbio, Beijing, China, 4091-99-0) was used to detect intracellular ROS levels. The cells were incubated with DCFH-DA working solution (5 μM) and 500 μL of the above regulated concentration cell suspension in the dark for 30 min. Then, the cell pellets were resuspended with 500 μL PBS at 300 g centrifugation at 4 °C for 5 min. FlowJo software was used to analyze the fluorescence intensity.

### 2.8. Cell Viability

The cells were seeded into 96-well plates for culture and various treatments. In the cell viability assay, cells were co-incubated with 10 μL of CCK8 reagent (Beyotime, Shanghai, China, C0038) for 2 h at 37 °C and absorbance was read at 450 nm with a microplate reader.

### 2.9. Statistical Analysis

Each of the experiments described here was performed in at least three independent biological replicates. GraphPad Prism 8.0 software was used for data analysis. Data are presented as mean ± standard error of mean (SEM). Statistical significance was determined by *t*-tests (two-tailed) for two groups or one-way ANOVA with Dunnett’s multiple comparisons test for more groups. Differences with *p* < 0.05 were considered significant.

## 3. Results

### 3.1. Effects of Eugenol on Serum Antioxidant Indicators in TGEV-Infected Weaned Piglets

**Figure 1:**
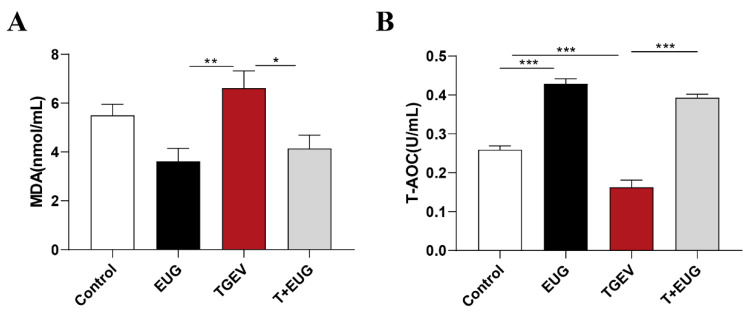
Effects of eugenol on serum antioxidant indicators in TGEV-infected weaned piglets. (**A**,**B**) The levels of MDA and T-AOC in serum (*n* = 8). Data are expressed as the mean ± SEM. * *p* < 0.05, ** *p* < 0.01, *** *p* < 0.001.

As shown in Figure 1A,B, eugenol supplementation significantly increased serum T-AOC level, and TGEV infection significantly reduced serum T-AOC level. In addition, under the condition of TGEV infection, eugenol supplementation significantly reduced serum MDA and increased serum T-AOC content.

### 3.2. Effects of Eugenol on Jejunum Antioxidation-Related Genes in TGEV-Infected Weaned Piglets

**Figure 2:**
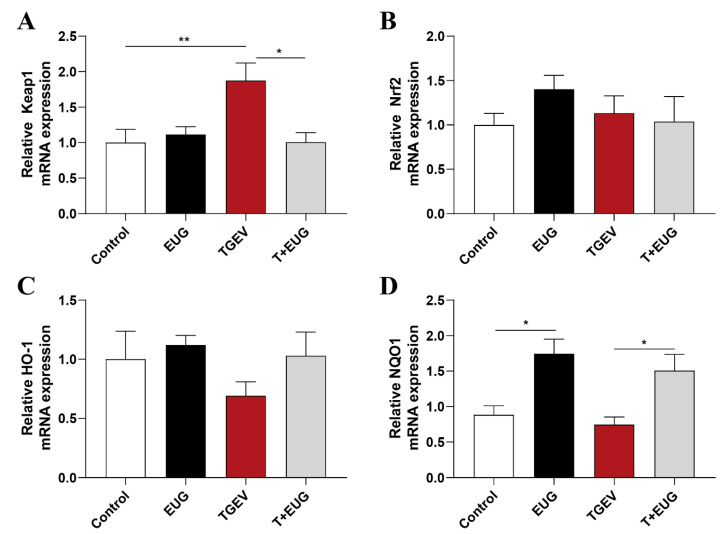
Effects of eugenol on jejunum antioxidation-related genes in TGEV-infected weaned piglets. (**A**–**D**) Relative mRNA expressions of Keap1, Nrf2, HO-1 and NQO1 in the jejunum mucosa (*n* = 6–8). Data are expressed as the mean ± SEM. * *p* < 0.05, ** *p* < 0.01.

Keap1-Nrf2-ARE signaling plays an important role in protecting cells from endogenous and exogenous stresses [28]. As shown in Figure 2A–D, TGEV infection increased the mRNA expressions of Keap1 in jejunum of weaned piglets, and eugenol supplementation increased the mRNA expressions of NQO1 in jejunum of weaned piglets. Of note, eugenol supplementation increases the mRNA expressions of NQO1 in jejunum of TGEV-infected piglets and alleviates the increase in Keap1 mRNA relative content.

### 3.3. Effects of Eugenol on Jejunum Antioxidation-Related Proteins in TGEV-Infected Weaned Piglets

**Figure 3:**
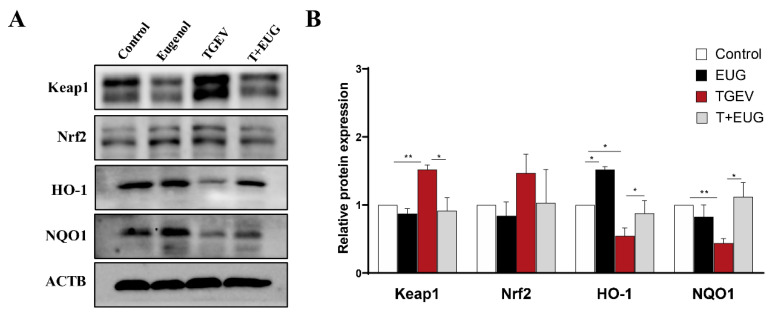
Effects of eugenol on jejunum antioxidation-related protein in TGEV-infected weaned piglets. (**A**,**B**) Protein abundance of Keap1, Nrf2, HO-1 and NQO1 in jejunum mucosa (*n* = 3). Data are expressed as the mean ± SEM. * *p* < 0.05, ** *p* < 0.01.

As shown in Figure 3A,B, TGEV infection significantly decreased the expression levels of HO-1 and NQO1 protein and increased the expression level of Keap1 protein in jejunum. In addition, eugenol supplementation significantly increased the TGEV-induced decrease in HO-1 and NQO1 protein levels and decreased the TGEV-induced increase in Keap1 protein level in jejunum.

### 3.4. Eugenol Decreases TGEV-Induced ROS Increase in Jejunal Epithelial Cells of Weaned Piglets

**Figure 4:**
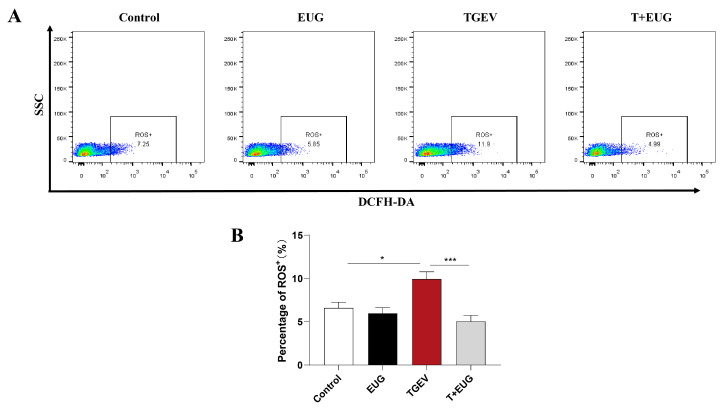
Eugenol decreases TGEV-induced ROS increase in jejunal epithelial cells of weaned piglets. (**A**,**B**) ROS levels in jejunum epithelial cells were analyzed by flow cytometry; SSC means side scatter (*n* = 6–8). Data are expressed as the mean ± SEM. * *p* < 0.05, *** *p* < 0.001.

Excessive production of ROS caused by abnormal redox regulation may be one of the causes of cell and tissue damage [29]. As shown in Figure 4A,B, notably, TGEV infection increased ROS level in the jejunum epithelial cells of weaned piglets. In addition, eugenol reduced ROS level in the jejunum epithelial cells of piglets induced by TGEV.

### 3.5. Eugenol Alleviates TGEV-Induced Jejunal Epithelial Cell Death in Piglets

**Figure 5:**
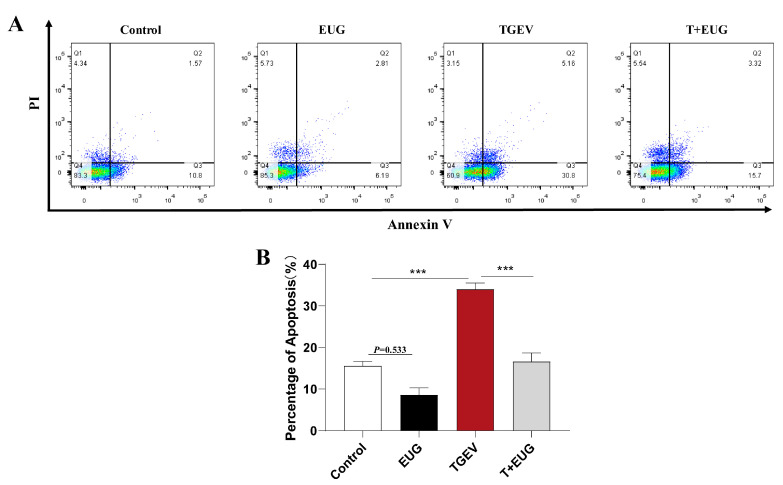
Eugenol alleviates TGEV-induced jejunal epithelial cell death in piglets. (**A**,**B**) The flow cytometry assays detecting the percentage of apoptosis in jejunum epithelial cells (*n* = 6–8). Data are expressed as the mean ± SEM. *** *p* < 0.001.

Apoptosis is a programmed series of events dependent on energy [30]. As shown in Figure 5A,B, notably, TGEV infection increased the proportion of apoptosis in jejunum epithelial cells of weaned piglets, and eugenol supplementation tended to decrease the proportion of apoptosis. Interestingly, eugenol supplementation significantly alleviated the increase in jejunum epithelial cell apoptosis induced by TGEV infection.

### 3.6. TGEV Damages the Antioxidant Capacity of IPEC-J2 Cells

**Figure 6:**
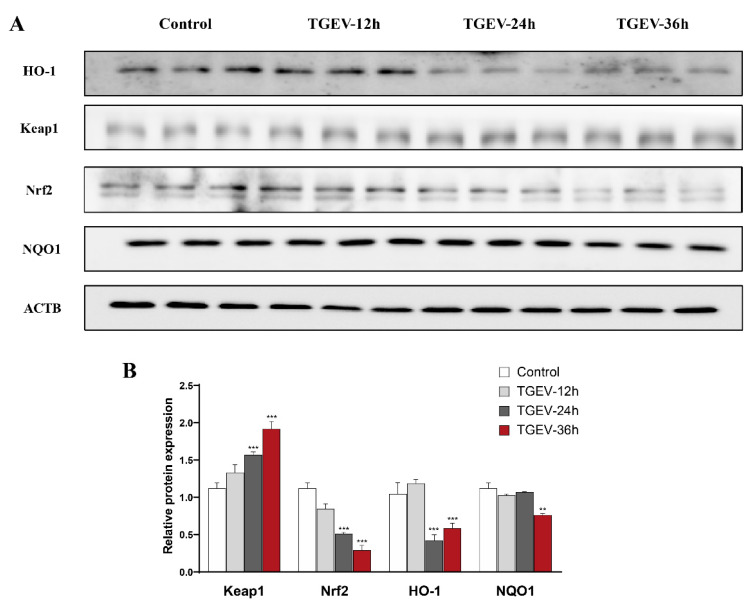
TGEV damages the antioxidant capacity of IPEC-J2 cells. (**A**,**B**) Protein abundance of Keap1, Nrf2, HO-1 and NQO1 in IPEC-J2 cells (*n* = 3). Data are expressed as the mean ± SEM. ** *p* < 0.01, *** *p* < 0.001.

To reveal the underlying mechanism by which eugenol attenuates TGEV infection in piglets, we further used IPEC-J2 cells infected with TGEV to construct an in vitro model. As shown in Figure 6A,B, with immunoblotting analysis, TGEV infection significantly increased Keap1 expression and decreased Nrf2, Ho-1 and NQO1 expression in IPEC-J2 cells for 36 h.

### 3.7. Effect of Eugenol on IPEC-J2 Cells Viability

**Figure 7:**
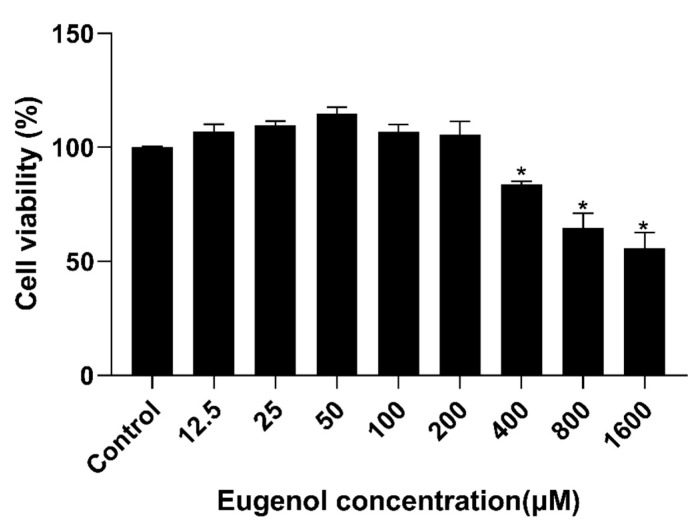
Effect of eugenol on IPEC-J2 cells’ viability. IPEC-J2 cells were treated with eugenol for 36 h (*n* = 6). Data are expressed as the mean ± SEM. * *p* < 0.05.

As shown in Figure 7, after IPEC-J2 cells were treated with different levels of eugenol for 36 h, high concentrations (400, 800 and 1600 μM) of eugenol significantly reduced IPEC-J2 cells viability. Therefore, eugenol with a concentration of 200 μM was selected for follow-up study.

### 3.8. Eugenol Alleviates TGEV-Induced Oxidative Stress in IPEC-J2 Cells

**Figure 8:**
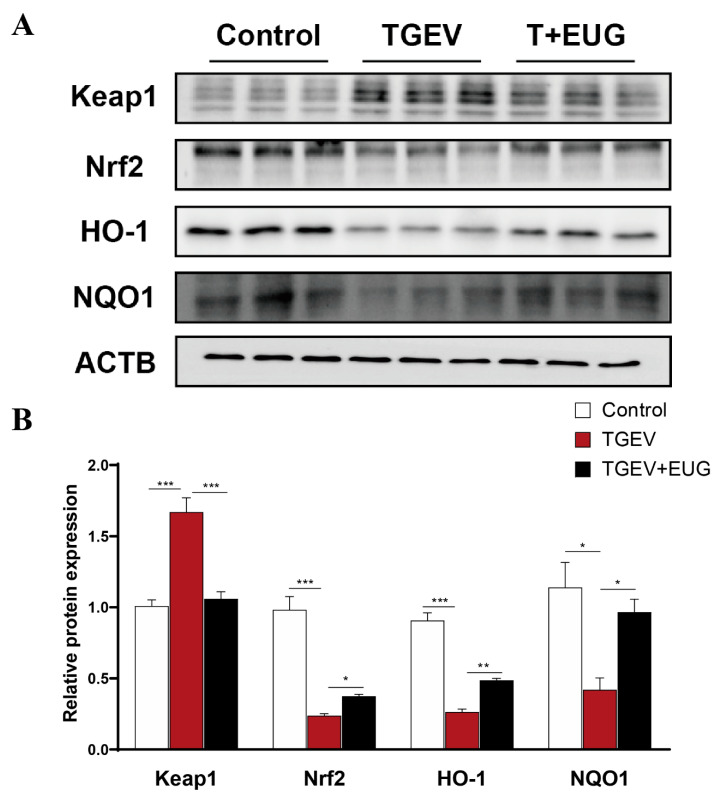
Eugenol ameliorates TGEV-induced oxidative stress in IPEC-J2 cells. (**A**,**B**) Protein abundance of Keap1, Nrf2, HO-1 and NQO1 in IPEC-J2 cells (*n* = 3). Data are expressed as the mean ± SEM. * *p* < 0.05, ** *p* < 0.01, *** *p* < 0.001.

We then explored the role of eugenol in TGEV-infected IPEC-J2 cells. As shown in Figure 8A,B, eugenol alleviated the impairment of TGEV infection on Keap1-Nrf2-ARE signaling in IPEC-J2 cells. With immunoblotting analysis, eugenol significantly decreased Keap-1 expression and significantly increased Nrf2, HO-1 and NQO1 expression in TGEV-infected IPEC-J2 cells.

### 3.9. Eugenol Relieves Oxidative Stress by Removing ROS

**Figure 9:**
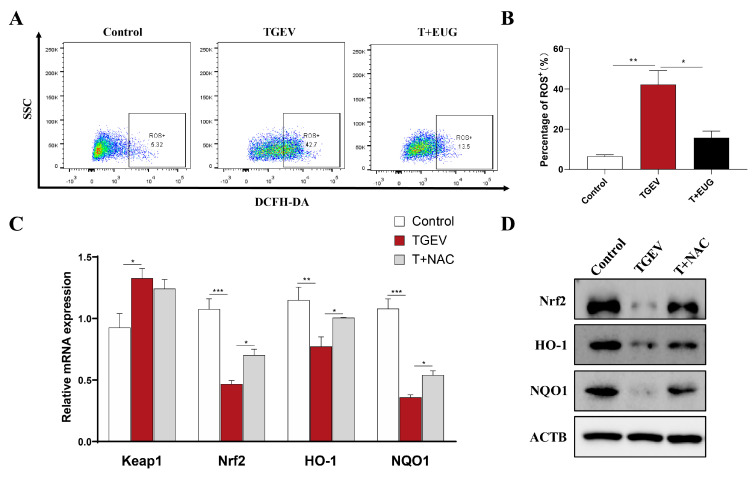
Eugenol relieves oxidative stress by removing ROS. IPEC-J2 cells were pretreated with eugenol (200 μM) or NAC (5 mM) for 1 h and then infected with TGEV for 36 h. (**A**,**B**) The flow cytometry assays detecting ROS levels in IPEC-J2 cells; SSC means side scatter (*n* = 3). (**C**) The expression of Keap1, Nrf2, HO-1 and NQO1 mRNA in IPEC-J2 cells were examined by RT-PCR (*n* = 3). (**D**) Protein abundance of Nrf2, HO-1 and NQO1 in IPEC-J2 cells. Data are expressed as the mean ± SEM. * *p* < 0.05, ** *p* < 0.01, *** *p* < 0.001.

As shown in Figure 9A,B, we found that eugenol significantly reduced ROS in TGEV-infected IPEC-J2 cells by flow cytometry. ROS play a key role in regulating redox signaling pathways [31]. Therefore, we used NAC (ROS scavenger) to further explore the mechanism by which eugenol alleviates TGEV-induced oxidative stress. NAC pretreatment of IPEC-J2 cells for 1 h significantly alleviated the TGEV-induced reduction in Nrf2, HO-1 and NQO1 mRNA expression levels (Figure 9C). However, NAC had no effect on the TGEV-induced increase in Keap1 mRNA levels. At the same time, we found that NAC treatment increased Nrf2, HO-1 and NQO1 protein expression levels in TGEV-infected IPEC-J2 cells (Figure 9D).

### 3.10. Effect of Eugenol on TGEV-Induced IPEC-J2 Cells’ Death Pattern

**Figure 10:**
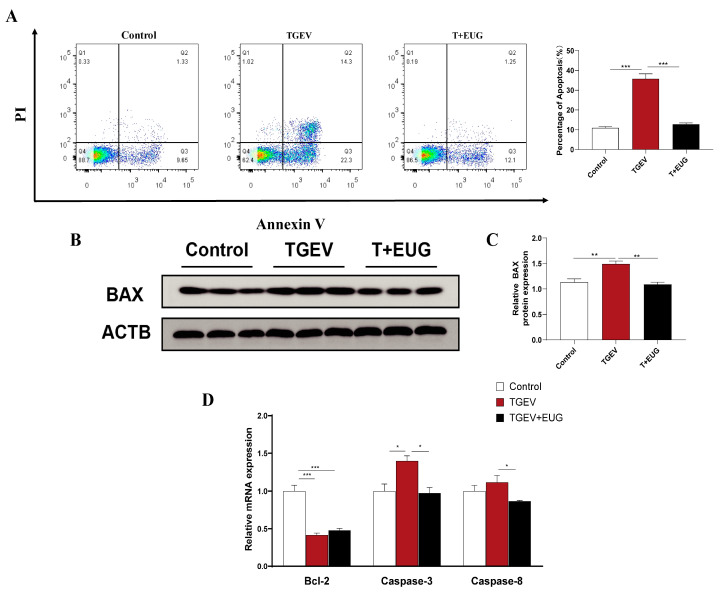
Effect of eugenol on TGEV-induced IPEC-J2 cells’ death pattern. IPEC-J2 cells were treated with 200 μM eugenol for 1 h and then infected with TGEV for 36 h. (**A**) The percentage of apoptosis in jejunum epithelial cells was analyzed by flow cytometry (*n*=3). (**B**,**C**) Immunoblot analysis and quantification of BAX in IPEC-J2 cells (*n* = 3). (**D**) Relative mRNA expressions of Bcl-2, Caspase-3 and Caspase-8 in IPEC-J2 cells (*n* = 3). Data are expressed as the mean ± SEM. * *p* < 0.05, ** *p* < 0.01, *** *p* < 0.001.

Apoptosis, a common mechanism of cell death, is currently the most extensively studied form. As shown in Figure 10A, TGEV infection increased the proportion of apoptosis (Q3), while eugenol treatment reduced TGEV-induced apoptosis. In addition, eugenol treatment significantly alleviated the TGEV-induced increase in Bax protein expression level and caspase-3 mRNA expression level, and decreased caspase-8 mRNA expression level, but had no effect on Bcl-2 mRNA expression level (Figure 10B,D).

## 4. Discussion

Under normal physiological conditions, ROS is produced at low levels and controlled by endogenous antioxidants [32]. T-AOC is the sum of enzymatic and non-enzymatic antioxidants. The T-AOC value increases with the increase in antioxidant capacity and decreases with the increase in lipid peroxidation. Therefore, T-AOC can reflect the antioxidant capacity and the level of oxidative damage [33]. MDA is a toxic end product of lipid peroxidation. Its expression level can directly reflect the rate and extent of lipid peroxidation, and indirectly reflects the ability to scavenge free radicals [34]. In the present study, it was observed that TGEV challenge increased the level of ROS in the jejunum of piglets and decreased T-AOC in serum. Many studies have shown that ROS levels are involved in apoptosis, and play an important role in regulating apoptosis [35,36,37]. Therefore, we hypothesized that the increase in intestinal ROS levels induced by TGEV infection might be involved in the occurrence of intestinal apoptosis. This may be the reason why TGEV induces further oxidative stress in the piglet intestine, which has not been reported before. Eugenol has excellent antioxidant capacity and free radical scavenging properties [38,39], and its addition to the diet can overcome the production of ROS caused by TGEV infection. Interestingly, we found a tendency for eugenol to reduce the percentage of apoptosis compared to the control group. This somewhat suggests that the antioxidant capacity of eugenol makes the intestine of piglets less exposed to weaning stress. This may be the direct antioxidant effect of eugenol, or it may be indirectly achieved through antioxidant pathways such as inhibiting MDA levels and increasing T-AOC. More research is needed.

In common antioxidant pathways, Nrf2 is a transcription factor that regulates the nuclear transcription of many genes encoding antioxidant proteins that are required to eliminate oxidative stress [40]. Once activated, Nrf2 dissociates from the Keap1-Nrf2 binding and translocates into the nucleus, where it transactivates genes driven by antioxidant response elements such as NQO1 and HO-1 [41,42]. In this study, TGEV infection significantly increased mRNA expressions of Keap1 in the jejunum of weaned piglets; significantly decreased HO-1 and NQO1 protein expression; and increased Keap1 protein expression in the jejunum of weaned pigs (which may be the indirect reason for the increase in ROS level induced by TGEV). In addition, under TGEV infection, the relative expression of NQO1 mRNA in the jejunum of piglets was significantly increased by dietary eugenol supplementation, which significantly alleviated the TGEV-induced decrease in HO-1 and NQO1 protein levels and the increase in Keap1 protein levels. We noted differences in mRNA and protein expression between HO-1 and NQO1 in the animal samples. The possible reasons may be as follows: (i) The homogeneity between samples is not good and the follow-up experiment can increase the sample size. (ii) Gene mRNA expression is not necessarily linearly related to protein expression because there are many factors regulating gene expression, and the regulation of transcription level is only one link. Post-transcriptional regulation and translational and post-translational regulation all have an impact on the final protein expression. (iii) Moreover, mRNA degradation, protein degradation, modification and folding may lead to inconsistency between mRNA expression and protein expression levels. In general, eugenol could prevent TGEV-induced ROS overproduction and oxidative stress-mediated intestinal apoptosis by enhancing T-AOC, NQO1 and HO-1 levels and decreasing MDA and Keap1 levels.

Disturbed redox homeostasis is a common feature of intestinal disease, characterized by uncontrolled ROS levels and impaired antioxidant defenses [8]. In general, viruses can benefit from either activating or inhibiting Nrf2 in host cells [43,44]. Our results demonstrate that TGEV infection disrupts the Keap1-Nrf2-ARE antioxidant defense system in IPEC-J2 cells. At the same time, eugenol alleviated the TGEV-induced increase in the protein expression level of Keap-1 in IPEC-J2 cells and ameliorated the TGEV-induced decrease in Nrf2, HO-1 and NQO1 protein expression in IPEC-J2 cells. We used NAC as a scavenger of ROS to gain insight into the mechanism by which eugenol alleviates TGEV-induced oxidative stress. A previous study showed that ROS levels affect the Keap1-Nrf2-ARE signaling pathway, which is consistent with our findings [45]. Our data demonstrated that the mRNA and protein expression of Nrf2, HO-1, and NQO1 were increased in IPEC-J2 cells after ROS scavenging in the presence of TGEV infection. This suggests that eugenol may improve the cellular antioxidant capacity by scavenging ROS, which is associated with its potent antioxidant capacity.

During viral infection, viruses interfere with many cellular functions by affecting various intracellular mediators, especially various major cell death pathways [46,47]. The dynamic balance of cell death mode is the functional basis for maintaining the continuous self-renewal of the body [48]. It is well known that the mitochondrial pathway is an important mechanism of apoptotic cell death. The mitochondrial pathway activates apoptotic factors Caspase-9, -8 and -3 [49], and is regulated by Bcl-2 family proteins [50]. TGEV infection simultaneously mediates PK-15 apoptosis through intrinsic and extrinsic apoptotic pathways [51]. Our results showed that eugenol supplementation alleviated TGEV-induced increases in Caspase-3 and Caspase-8 mRNA, as well as BAX protein expression in IPEC-J2 cells. In parallel, the flow cytometry results showed that eugenol downregulated the apoptotic rate of TGEV-infected IPEC-J2 cells. It is noteworthy that ROS are involved in virus-induced apoptosis [52,53,54]. In addition, porcine parvovirus infection leads to ST cell apoptosis through the activation of the ROS-mediated mitochondrial apoptosis pathway [55]. These results imply that eugenol alleviates the apoptosis of IPEC-J2 cells, which may be related to the clearance of intracellular ROS and the enhancement of antioxidant capacity.

The effect of oxidative stress is not specific to TGEV. According to relevant literature, other coronaviruses (SARS-CoV [56], SARS-CoV-2 [57,58] and MERS [59]) can also induce oxidative stress. However, there are no relevant reports showing the effect of eugenol on other coronavirus. Recent reports suggest that TGEV can provide a good model for human highly pathogenic coronaviruses [60,61]. In addition, oxidative stress is one of the pathogeneses of aging, diabetes and cancer. Therefore, we believe that eugenol, a natural product with antioxidative stress properties, has the potential to be an antiviral, antiaging, antidiabetic and anticancer medication.

## 5. Conclusions

In conclusion, our results reveal the potential therapeutic use of eugenol in TGEV infection. We confirmed that eugenol alleviates TGEV-induced oxidative stress in intestinal epithelial cells by regulating ROS, thereby affecting the Keap1-Nrf2-ARE signaling pathway and reducing TGEV-induced excessive cell apoptosis. This study may reveal the novel mechanisms by which eugenol alleviates oxidative stress in TGEV infection and contribute to its future application in coronavirus-related diseases.

## Figures and Tables

**Table 1:** Composition and nutrient level of basal diet.

Ingredients	%	Nutrient Level ^3^	Contents
Corn	33.80	Digestible energy (calculated, Mcal/kg)	3.54
Extruded corn	22.20	Crude Protein (%)	19.49
Soybean meal	7.42	Calcium (%)	0.75
Extruded full-fat soybean	8.79	Available phosphorus (%)	0.37
Fish meal	3.94	Lysine	1.35
Whey powder	5.00	Methionine	0.39
Soybean protein concentrate	8.00	Methionine + cysteine	0.68
Soybean oil	1.65	Threonine	0.80
Sucrose	2.00	Tryptophan	0.22
Limestone	0.62		
Dicalcium phosphate	0.46		
NaCl	0.20		
L-Lysine HCl (78%)	0.32		
DL-Methionine	0.07		
L-Threonine (98.5%)	0.02		
Tryptophan (98%)	0.01		
Chloride choline	0.15		
Vitamin premix ^1^	0.05		
Mineral premix ^2^	0.30		
Total	100		

^1^ The vitamin premix provided the following per kg of diet: 6000 IU of VA, 3000 IU of VD_3_, 24 IU of VE, 3 mg of VK_3_, 1.5 mg of VB_1_, 6 mg of VB_2_, 3 mg of VB_6_, 0.02 mg of VB_12_, 14 mg of niacin, 15 mg of pantothenic acid, 0.75 mg of folic acid, and 0.1 mg of biotin. ^2^ The mineral premix provided the following per kg of diet: Fe(FeSO4·H2O), 100 mg; Cu(CuSO_4_·5H_2_O), 6 mg; Mn(MnSO_4_·H_2_O), 4 mg; Zn(ZnSO4·H2O), 100 mg; I(KI), 0.3 mg; Se(Na_2_SeO_3_), 0.3 mg. ^3^ Nutrient level values are calculated.

**Table 2:** Primer sequences table.

Gene	Primers	Sequences	Product size	Accession Numbers
*β-actin*	Forward	GCAAATGCTTCTAGGCGGAC	148	XM_021086047.1
	Reverse	GCGTCCATCACAGCTTCTCA		
*Keap1*	Forward	TCTGCTTAGTCATGGTGACCT	143	NM_001114671.1
	Reverse	AAGGGACAACACCACCACTG		
*Nrf2*	Forward	CTACGGGATTGGGGTTTGGG	124	XM_013984303.2
	Reverse	AACTCAAACAGGGGAAGGGC		
*HO-1*	Forward	TACCGCTCCCGAATGAACAC	140	NM_001004027.1
	Reverse	TGGTCCTTAGTGTCCTGGGT		
*NQO1*	Forward	TGCTTACACATACGCTGCCA	113	NM_001159613.1
	Reverse	CGTGGATACCCTGCAGAGAG		
*Bcl-2*	Forward	AGCATGCGGCCTCTATTTGA	120	XM_021099593.1
	Reverse	GGCCCGTGGACTTCACTTAT		
*Caspase-3*	Forward	GGATTGAGACGGACAGTGGG	124	NM_214131.1
	Reverse	CCGTCCTTTGAATTTCGCCA		
*Caspase-8*	Forward	GGATCCCAGGATTTGCCTCC	135	NM_001031779.2
	Reverse	CAGGCTCAGGAACTTGAGGG		

## Data Availability

The original contributions presented in the study are included in the article. Further inquiries can be directed to the corresponding author.
